# HIV virological non-suppression is highly prevalent among 18- to 24-year-old youths on antiretroviral therapy at the Kenyan coast

**DOI:** 10.1186/s12879-022-07428-w

**Published:** 2022-05-11

**Authors:** Moses K. Nyongesa, Mwaganyuma H. Mwatasa, Vincent A. Kagonya, Gabriel Mwambingu, Caroline Ngetsa, Charles R. J. C. Newton, Amina Abubakar

**Affiliations:** 1 KEMRI-Wellcome Trust Research Programme, Centre for Geographic Medicine Research (Coast), Box 230, Kilifi, Kenya; 2 Department of Clinical, Neuro- and Developmental Psychology, Amsterdam Public Health Research Institute, Vrije Universiteit Amsterdam, Amsterdam, The Netherlands; 3 Department of Public Health, Pwani University, Kilifi, Kenya; 4 Department of Psychiatry, University of Oxford, Oxford, UK; 5 Institute for Human Development, Aga Khan University, Nairobi, Kenya

**Keywords:** HIV infections, Antiretroviral therapy, Virological non-suppression, Prevalence, Young people, Youths living with HIV, Risk indicators, Kenya

## Abstract

**Background:**

In sub-Saharan Africa, data on virologic outcomes of young people living with HIV (YLWH) enrolled on antiretroviral therapy (ART) remains scarce. In this study, we describe the prevalence of HIV virological non-suppression (VNS) and its associated factors among YLWH aged 18–24 years from the Kenyan coast.

**Methods:**

Data were analyzed for 384 YLWH who participated in a larger cross-sectional study conducted between November 2018 and September 2019 in two counties at the Kenyan coast (Kilifi and Mombasa). Descriptive statistics were used to summarize sample characteristics and logistic regression was used for statistical modeling of factors associated with VNS. In this study, VNS was defined as plasma viral load ≥ 1000 copies/mL.

**Results:**

Among these YLWH with a mean age of 20.7 years (SD = 2.2); 55.5% females, the overall prevalence of VNS was *32.0%* (*95% Confidence interval *(*95% CI*)*: 27.5, 36.9%*). In the multivariable logistic regression analysis, being from a largely rural setting (*adjusted Odds Ratio *(*aOR*)* 1.73, 95% CI 1.10, 2.71; p* = *0.02*), underweight (*aOR 1.87, 95% CI 1.16, 3.01; p* = *0.01*) and low self-reported ART adherence (*aOR 2.83, 95% CI 1.34, 6.00; p* = *0.01*) were significantly associated with higher odds of VNS in YLWH.

**Conclusions:**

In this study, high levels of VNS were observed among YLWH and this was significantly associated with rural residency, nutritional and ART adherence problems. ART adherence counselling and nutritional support and education should be intensified in this setting targeting YLWH residing mostly in rural areas. Given the high frequency of VNS, there is need to closely monitor viral load and profile HIV drug resistance patterns in youths from the Kenyan coast with confirmed virologic failure. The latter will help understand whether drug resistance also contributes to poor viral suppression in addition to, or exclusive of suboptimal ART adherence.

## Background

Globally, 3.4 million young people aged 15–24 years were living with HIV by 2019 [1]—majority reside in sub-Saharan Africa (SSA) [2]. The number of young people living with HIV (YLWH) continues to unprecedentedly increase as more become newly HIV-infected, mostly through sexual transmission [3]. Of the approximately 4500 new cases of HIV infection reported daily, about a third (31%) are among young people aged 15–24 years [4] with a large proportion being youths from SSA [5]. In SSA, out of every four reported new HIV infections, three occur in young people [6]. In Kenya, young people aged 15–24 years contributed over half of new adult HIV infections according to the 2015 estimates [7].

The advent, increasing access and continued adherence to antiretroviral therapy (ART) has transformed the management of HIV from a fatal disease to now a manageable chronic condition [8]. A key goal of ART is suppression of viral replication leading to preservation of an optimal state of health with improved immune functioning. In terms of HIV treatment outcomes, consistent use of ART has had substantial gains including declined HIV-related morbidity and mortality [9] and improved survival [10]. A threat to these gains is the poor adherence to ART observed among YLWH compared to younger children and adults [11–13]. Not only does this poor adherence place these youths at risk of HIV virological non-suppression (VNS) but also limits the efforts towards achieving the last two 95’s of the ambitious 95–95–95 HIV treatment targets by the year 2030 [14]. According to the World Health Organization (WHO) consolidated ART guidelines for a public health approach [15], an individual is considered to have VNS if their plasma viral (HIV-ribonucleic acid) load is ≥ 1000 copies/mL.

Studies from different geographical settings—mostly in the West—have investigated virologic suppression status among YLWH on ART. In general, suboptimal level of viral suppression among these youths is evident. In a global review of virological outcomes of YLWH [16], the prevalence of VNS ranged between 11 and 73%. In a review of South African studies looking at YLWH’s viral suppression among other aspects in the HIV continuum of care, Zanoni et al. [17] report VNS prevalence ranging from 7 to 27%. VNS can lead to adverse outcomes such as higher risk of onward HIV transmission (for those who engage in risky sexual behavior) [18], viral rebound [19], faster disease progression and increased morbidity and mortality [20]. Poor school attendance and lose of economic productivity have also been documented [21].

Investigations of the factors associated with VNS among YLWH are generally scarce, more so in SSA. Most of the existing studies are limited in terms of consensus and some studies present contradictory findings, albeit because of contextual differences. For instance, long duration on ART was found to be significantly associated with lower odds of VNS among Cambodian youths [22], while among Ethiopian youths [23] this was associated with significantly higher odds of VNS. In SSA, male sex [24, 25] and poor ART adherence [24, 26] appear important risk indicators for VNS in YLWH; there is some consensus between studies but more research is warranted for a clear insight. Other risk indicators for VNS reported by studies conducted in Ethiopia and Malawi include WHO HIV clinical stage 2 [24], HIV-related stigma [25], lower socioeconomic status and poor self-reported wellbeing [23]. Whether these are also important correlates of VNS among YLWH across SSA is inconclusive; further research evidence is needed from other SSA countries.

Data on virologic outcomes of YLWH enrolled on ART in SSA is limited. In particular, knowledge about the factors associated with VNS in this sub-population is not widely documented. In Kenya, we are aware of a recent study investigating VNS among YLWH 10–24 years old [27] but this study did not explore the factors independently associated with VNS and participants were enrolled from an urban setting only. Information on correlates of VNS is critical in informing potential intervention areas for optimization of ART outcomes among young people. In the present study, we: i) determined the prevalence of VNS among YLWH 18–24 years old enrolled from two diverse geographical settings at the Kenyan coast; and ii) investigated the factors associated with VNS among these young people.

## Methods

### Study design

This work is part of data collected from a larger cross-sectional study conducted between November 2018 and September 2019 along the Kenyan coast. The details of the methodology have been described previously [28]. Below, we provide a brief description of the study setting, participants and their recruitment process.

### Study setting, participants and recruitment

Study participants were YLWH recruited through consecutive sampling from 20 geographically diverse HIV-clinics in two counties at the Kenyan coast (Kilifi, n = 13 and Mombasa, n = 7). Kilifi County is largely a rural setting with an estimated population of 1.5 million people [29]. HIV prevalence among individuals aged 15 years or older in this County is estimated at 4.5%, slightly lower than the national average prevalence of 6% [30]. Mombasa County is adjacent to Kilifi County. It is an urban County hosting one of the three major cities of Kenya (Mombasa) and has an estimated population of 1.2 million people [29]. HIV prevalence among individuals aged 15 years or older in this County is estimated at 7.5% [30].

**Fig. 1:**
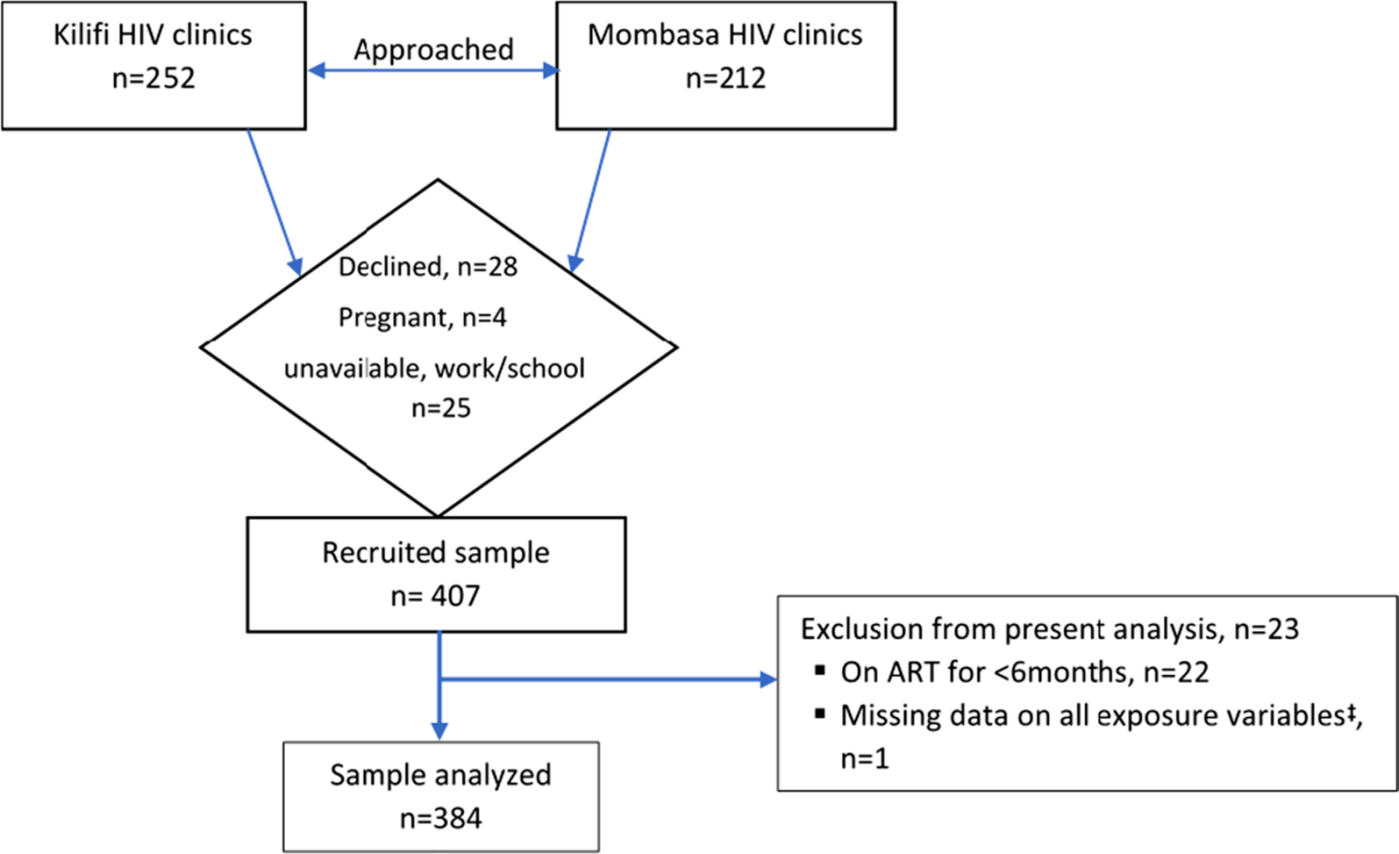
Recruitment flow chart for YLWH. ^**‡**^Due to a technical error on the electronic data capture platform, and participant could not be reached on contact details provided for another interview. Pregnancy was one of the exclusion criteria applied in the larger study

Eligibility criteria in the larger study included: age range of 18–24 years, a confirmed HIV-positive status, being on ART and providing informed consent for participation. YLWH were identified from existing clinic records, contacted, and invited for study briefs at a day coinciding with the monthly teen support group meetings. Those who could not be reached via available mobile contacts or without contact details were traced during their next scheduled clinic appointment dates. Bookings for study interviews were done after consenting participants had been taken through the study in details by research assistants present at the time of the meeting. Eligible for the present analysis were young people on ART for at least 6 months (see Fig. 1).

### Sample size calculation

The sample size was originally calculated during the design of the larger study based on key outcomes described elsewhere [28]. In the present analysis, the sample of 384 eligible participants is > 95% powered (at 5% level of significance) to detect a proportion of at least 0.20 given a null proportion of 0.11 [31]. The same sample is > 85% powered (at 5% level of significance) to carry out a logistic regression analysis of correlates of VNS based on previously reported effect sizes [23].

### Measures

### Outcome measure

#### Viral load quantification

Whole blood samples were collected from each of the participating YLWH by the study nurse trained in phlebotomy. At the Clinical Trials Laboratory of the KEMRI-Wellcome Trust Research Programme, plasma samples were extracted from each of the collected blood samples. HIV viral load was quantified using the GeneXpert® Dx System equipment (version 4.8; United States of America) utilizing about 1200 µL of plasma. All the remaining plasma were individually labelled and archived at − 80 °C for future investigations that will involve ART plasma concentration and HIV drug resistance genotyping. In the event the viral load test failed on the first attempt, an extra 1200µL of archived plasma was retrieved for a repeat run. In this study, YLWH were categorized as having VNS if their plasma viral load was ≥ 1000 copies/mL in line with WHO recommendations [15]. All the viral load results were also shared with the respective HIV clinics to facilitate continuity of treatment.

#### Measures of exposure variables

##### Sociodemographic and asset index forms

The sociodemographic form captured participant’s age, sex, educational level, employment status, religion, relationship status, area of residence, living status of parents and whom they currently lived with. The asset index form gathered information about individual or family ownership of disposable items as a proxy indicator of socioeconomic status.

##### Lifestyle and health history form

This form captured data on whether participants currently smoked cigarette, chewed khat or used alcohol using a yes/no response option via audio computer-assisted self-interview (ACASI). Self-report information sought from YLWH included disclosure of their HIV status, their level of satisfaction with current care and their view on accessibility of their current point-of-care. YLWH were also asked whether they experienced any current ART side effects or an opportunistic infection, as informed by their clinician.

##### HIV-related clinical data

This﻿ ﻿included data on participant current ART regimen and duration they have been on ART, WHO clinical staging of HIV, height and weight (recorded during the most recent clinic visit) for body mass index (BMI) computation, categorized as underweight (BMI < 18.5), normal (BMI of 18.5–24.9), overweight (BMI of 25.0–29.9) and obese (BMI ≥ 30.0). Participants were managed based on local guidelines on use of antiretroviral drugs for treating and preventing HIV infection [32].

##### Psychiatric symptoms

A locally validated version of *the 9-item patient health questionnaire (PHQ-9)* [33], administered via ACASI, was used to assess presence of depressive symptoms among the study participants. Items on this measure are rated on a 4-point Likert scale of “0” (not at all) to “3” (nearly every day). A total score ranging between 0 and 27 is derived from the summation of item scores. According to the scoring protocol [34], a cut-off score of ≥ 10, also applicable for the Eastern Africa setting [35], indicates positive screen for depressive symptoms.

*The brief 12-item HIV stigma scale* [36], administered via ACASI, assessed participants’ perceived HIV-related stigma. This scale is rated on a 4-point Likert scale as “1” (strongly disagree), “2” (disagree) “3” (agree) and “4” (strongly agree). Item scores are summated to derive a total score ranging between 12 and 48, higher scores indicative of a greater levels of perceived HIV-related stigma.

*The 4-item Morisky, Green and Levine Medication Adherence Scale *(*MGLS*) [37] was used as a self-report measure of ART adherence. This measure was also administered via ACASI. The MGLS has a total score ranging between 0 and 4. Based on this score, there are 3 levels of medication adherence: high (a score of 0), medium (scores of 1 or 2), and low adherence (scores ≥ 3).

### Statistical analysis

All analyses were conducted in STATA version 15.0 (StataCorp LP, College Station, Texas, United States of America). Basic descriptive statistics (means [SD], frequencies and percentages) were used to summarize sample characteristics by group viral suppression status (virally suppressed vs. non-suppressed groups). Chi-squared (χ^2^) test and independent Student’s t-test were used to compare group differences for categorical and continuous variables, respectively. Proportions as percentages were used to estimate the prevalence of VNS. Univariate logistic regression analysis was used to assess the crude association between exposure variables (sociodemographic, psychosocial and HIV-related factors) and the outcome variable (VNS). All variables with p < 0.25 from univariate analyses were taken forward to the multivariable logistic regression analysis which was used to investigate factors independently associated with VNS. Backward stepwise logistic regression models were fit in the multivariable model building process, removing all variables with p > 0.05 (one at a time). Collinearity diagnostics were performed for the final multivariable model, with no multicollinearity problems identified based on interpretation of the variance inflation factor. A p-value > 0.05 in the Hosmer–Lemeshow goodness of fit test was considered a well-fitting final logistic regression model. For all tests of hypothesis, a two-tailed p-value of < 0.05 was considered statistically significant, with a confidence interval of 95% used to report on the precision of the reported estimates.

## Results

### Participant characteristics

Table 1 shows the participant sociodemographic, psychosocial and HIV-related characteristics presented by HIV viral load suppression status, alongside results from univariate logistic regression analyses. In summary, the mean age of the participants was 20.7 years (SD = 2.2), more than half (55.5%) were females. Over a quarter of the participants screened positive for depressive symptoms (27.9%). Most of the participants had a BMI within the normal range (59.9%), were on ART for over 5 years (61.2%), largely first line regimen (80.5%), and were highly adherent based on self-report (65.9%). Participants’ mean asset index and perceived HIV-related stigma scores were 2.2 (SD = 1.6) and 25.9 (SD = 7.4), respectively.

**Table 1:** Participant characteristics by HIV viral load suppression status and associated crude odds ratio

		Viral suppression status			Unadjusted OR	
Sociodemographic factors						
Area of residence	Urban	196 (51.0)	144 (55.2)	52 (42.3)	Ref	
	Rural	188 (49.0)	117 (44.8)	71 (57.7)	1.68 (1.09, 2.60)	0.02
Age (years)	Mean (SD)	20.7 (2.2)	20.7 (2.2)	20.7 (2.2)	0.99 (0.89, 1.09)	0.80
Sex	Female	213 (55.5)	150 (57.5)	63 (51.2)	Ref	
	Male	171 (44.5)	111 (42.5)	60 (48.8)	1.29 (0.84, 1.98)	0.25
Education level	None	7 (1.8)	6 (2.3)	1 (0.8)	Ref	
	Primary	151 (39.3)	106 (40.6)	45 (36.6)	2.55 (0.30, 21.77)	0.39
	Secondary	168 (43.8)	107 (41.0)	61 (49.6)	3.42 (0.40, 29.08)	0.26
	Tertiary	58 (15.1)	42 (16.1)	16 (13.0)	2.29 (0.25, 20.50)	0.46
Employment	Employed	53 (13.8)	37 (14.2)	16 (13.0)	Ref	
	Student	138 (35.9)	95 (36.4)	43 (35.0)	1.05 (0.53, 2.08)	0.90
	Unemployed	193 (50.3)	129 (49.4)	64 (52.0)	1.15 (0.59, 2.22)	0.68
Religion	No religion	12 (3.1)	9 (3.5)	3 (2.4)	Ref	
	Christian	287 (74.7)	195 (74.7)	92 (74.8)	1.42 (0.37, 5.35)	0.61
	Muslim	85 (22.1)	57 (21.8)	28 (22.8)	1.47 (0.37, 5.87)	0.58
Relationship status	With a partner^1^	64 (16.7)	49 (18.8)	15 (12.2)	Ref	
	No partner^2^	320 (83.3)	212 (81.2)	108 (87.8)	1.66 (0.89, 3.10)	0.11
Living arrangement	Living alone	34 (8.9)	27 (10.3)	7 (5.7)	Ref	
	Family/relative	343 (89.3)	230 (88.1)	113 (91.9)	1.90 (0.80, 4.48)	0.15
	Friend/other	7 (1.8)	4 (1.5)	3 (2.4)	2.89 (0.52, 16.03)	0.22
Asset index score^a^	Mean (SD)	2.2 (1.6)	2.2 (1.6)	2.3 (1.7)	1.06 (0.93, 1.21)	0.38
HIV-related factors						
Body Mass Index	Normal	230 (59.9)	164 (62.8)	66 (53.7)	Ref	
	Overweight	26 (6.8)	19 (7.3)	7 (5.7)	0.92 (0.37, 2.28)	0.85
	Obese	13 (3.4)	12 (4.6)	1 (0.8)	0.21 (0.03, 1.62)	0.13
	Underweight	115 (30.0)	66 (25.3)	49 (39.8)	1.84 (1.16, 2.94)	0.01
WHO HIV clinical stage (missing = 1)	Stage 1 & Stage 2	330 (86.2)	225 (86.5)	105 (85.4)	Ref	
	Stage 3 & Stage 4	53 (13.8)	35 (13.5)	18 (14.6)	1.10 (0.60, 2.04)	0.76
Current ART regimen	First line	309 (80.5)	212 (81.2)	97 (78.9)	Ref	
	Second line	75 (19.5)	49 (18.8)	26 (21.1)	1.16 (0.68, 1.98)	0.59
Duration on ART	< 1 year	25 (6.5)	21 (8.0)	4 (3.3)	Ref	
	1–5 years	124 (32.3)	90 (34.5)	34 (27.6)	1.98 (0.63, 6.20)	0.24
	> 5 years	235 (61.2)	150 (57.5)	85 (69.1)	2.97 (0.99, 8.95)	0.05
Any ART side effects (missing = 1)	No	279 (72.9)	187 (71.9)	92 (74.8)	Ref	
	Yes	104 (27.1)	73 (28.1)	31 (25.2)	0.86 (0.53, 1.41)	0.56
ART Adherence	High adherence	238 (62.0)	172 (65.9)	66 (53.7)	Ref	
	Medium	111 (28.9)	71 (27.2)	40 (32.5)	1.47 (0.91, 2.37)	0.12
	Low	35 (9.1)	18 (6.9)	17 (13.8)	2.46 (1.20, 5.06)	0.01
Any current opportunistic infection	No	360 (93.7)	251 (96.2)	109 (88.6)	Ref	
	Yes	24 (6.3)	10 (3.8)	14 (11.4)	3.22 (1.39, 7.48)	0.01
Psychosocial factors						
Parental loss	All alive	115 (30.0)	84 (32.2)	31 (25.2)	Ref	
	One parent alive	141 (36.7)	87 (33.3)	54 (43.9)	1.68 (0.99, 2.87)	0.06
	Both Died	128 (33.3)	90 (34.5)	38 (30.9)	1.14 (0.65, 2.00)	0.64
Disclosure of HIV status	No	19 (5.0)	18 (6.9)	1 (0.8)	Ref	
	Yes, family only	312 (81.2)	208 (79.7)	104 (84.6)	9.00 (1.19, 68.35)	0.03
	Yes, very open	53 (13.8)	35 (13.4)	18 (14.6)	9.26 (1.14, 75.02)	0.04
Clinic accessibility	< 30 min travel time	123 (32.0)	86 (33.0)	37 (30.1)	Ref	
	30 min–1 h	166 (43.2)	112 (42.9)	54 (43.9)	1.12 (0.68, 1.85)	0.66
	> hour travel time	95 (24.7)	63 (24.1)	32 (26.0)	1.18 (0.66, 2.10)	0.57
Satisfaction with current care	Satisfied	364 (94.8)	246 (94.3)	118 (95.9)	Ref	
	Unsatisfied	20 (5.2)	15 (5.7)	5 (4.1)	0.69 (0.25, 1.96)	0.49
Currently smoking	No	367 (95.6)	250 (95.8)	117 (95.1)	Ref	
	Yes	17 (4.4)	11 (4.2)	6 (4.9)	1.17 (0.42, 3.23)	0.77
Current use of khat	No	355 (92.4)	245 (93.9)	110 (89.4)	Ref	
	Yes	29 (7.6)	16 (6.1)	13 (10.6)	1.81 (0.84, 3.89)	0.13
Current alcohol use	No	334 (87.0)	232 (88.9)	102 (82.9)	Ref	
	Yes	50 (13.0)	29 (11.1)	21 (17.1)	1.65 (0.90, 3.03)	0.11
Depressive symptoms	No	277 (72.1)	196 (75.1)	81 (65.8)	Ref	
	yes	107 (27.9)	65 (24.9)	42 (34.2)	1.56 (0.98, 2.49)	0.06
Perceived HIV stigma	Mean (SD)	25.9 (7.4)	25.6 (7.6)	26.7 (6.9)	1.02 (0.99, 1.05)	0.17

All values are presented as frequency (percent) unless otherwise stated

^1^Included those who were married or cohabiting

^2^Included those who were never married, those widowed, divorced or separated

^a^Score range = 0 to 7, higher scores indicate better socioeconomic status

*SD* standard deviation, *CI* confidence interval, *OR* odds ratio, *HIV* human immunodeficiency virus, *ART* antiretroviral therapy, *WHO* World Health Organization

### Prevalence of VNS among YLWH on ART from the Kenyan coast

**Fig. 2:**
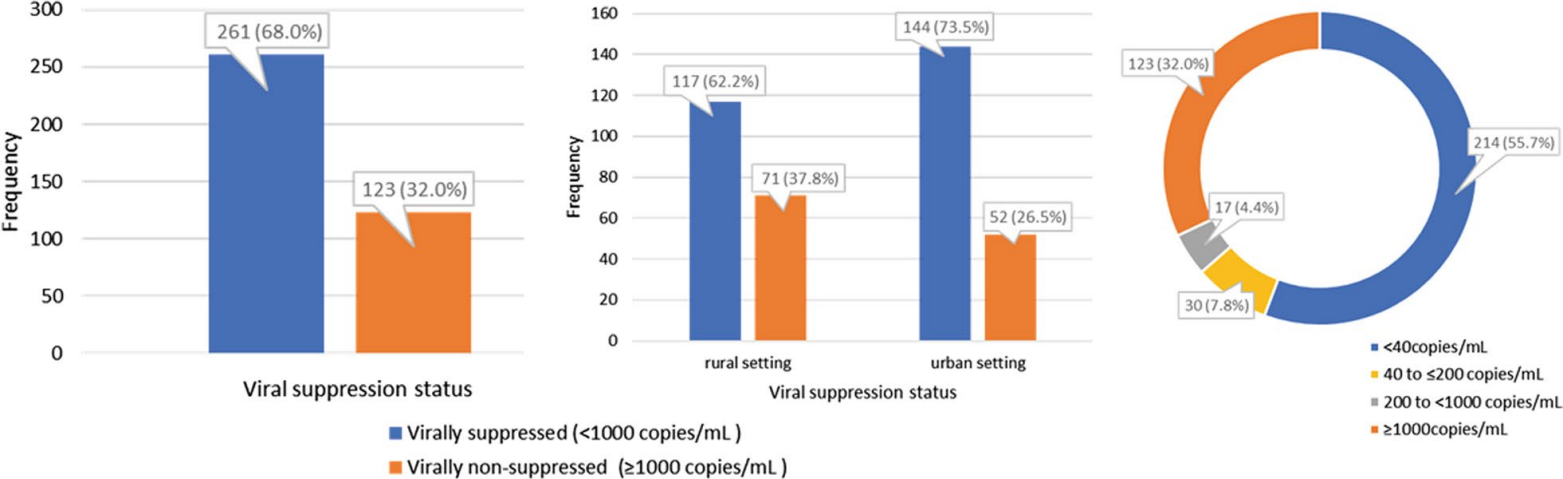
HIV viral load suppression status among young people, 18–24 years old, on ART from coastal Kenya, n = 384

Figure 2 depicts the HIV viral load suppression status among young people on ART from the Kenyan coast. Most of these young people (56%) had undetectable HIV viral load (< 40copies/mL). The overall prevalence of VNS was high**,** *32.0% *(*95% Confidence interval *(*95% CI*)*: 27.5, 36.9%*). The prevalence of VNS was significantly higher among YLWH from the rural setting compared to those from the urban setting, *37.8% *(*95% CI 30.8, 44.7%*)* vs 26.5% *(*95% CI 20.3, 32.7%*)*; p* = *0.02*.

### Factors associated with VNS among young people from coastal Kenya

The univariate logistic regression analysis showed that being from a rural setting, underweight, low self-reported ART adherence, presence of a current opportunistic infection and disclosure of HIV status significantly increased the odds of VNS (Table 1). Lack of a partner, living with others, being obese, being on ART for a year or more, medium ART adherence, loss of one parent, current khat use, presence of depressive symptoms and higher perceived HIV-related stigma were associated with VNS at p < 0.25 (Table 1) and were all included in the multivariable logistic regression analysis.

Under the final multivariable logistic regression analysis, being from a rural setting (*aOR 1.73 95% CI 1.10, 2.71; p* = *0.02*), underweight (*aOR 1.87 95% CI 1.16, 3.01; p* = *0.01*) and low self-reported adherence to ART (*aOR 2.83 95% CI 1.34, 6.00; p* = *0.01*) were significantly associated with higher odds of VNS. Hosmer–Lemeshow goodness of fit statistic for the final regression model was *X*^*2*^_(*15*)_ = *9.97; p* = *0.82*.

## Discussion

In this cross-sectional study from the Kenyan coast, we describe the prevalence and factors associated with VNS among YLWH aged 18–24 years. Almost a third (32%) of these youths were virally non-suppressed and this was significantly associated with rural residence, being underweight and low ART adherence. Other studies investigating VNS among YLWH in SSA and beyond report varying prevalence estimates, some similar to what we observed, for instance estimates of 26% reported by a study conducted in Ethiopia [24], 37% in South Africa [19], 39% in Malawi [25], 40% in Tanzania [38], and 23% in Cambodia [22]. We found VNS prevalence of 27% among participants from the urban setting which compares to the VNS prevalence of 26% reported by Kangethe et al. [27] in their study involving YLWH recruited from a tertiary facility in Kenya’s capital city of Nairobi.

Across the above studies documenting VNS prevalence, of note is the differences in the threshold applied when defining VNS and the recruited age groups of YLWH which may account for the variation in prevalence estimates. For instance, the South African [19] and Tanzanian [38] studies may have recorded estimates on the higher side because of using a much lower threshold to define VNS i.e. plasma viral load ≥ 400 copies/mL. The rest of the studies applied a threshold similar to our study i.e. plasma viral load ≥ 1000 copies/mL. However, the Malawian study [25] recruited a broader age group of 13 to 24-year-old YLWH whereas the Cambodian study [22] recruited a narrower age group of 15 to 17-year-old YLWH. This, in addition to contextual differences may explain the extreme ends of observed VNS prevalence in these two studies (39% vs. 23%). At the Kenyan coast, the observed prevalence of VNS among YLWH aged 18–24 years is higher than the 25% prevalence estimate reported by Hassan et al. [39] among the general adult population living with HIV. These authors used a threshold of plasma viral load ≥ 400 copies/mL to define VNS. Arguably, their observed prevalence estimate would have been lower if a much higher threshold were applied.

As would be expected and consistent with previous findings [24, 26, 39, 40], the odds of VNS were significantly higher among participants who self-reported low ART adherence. Since excellent ART adherence mediates the relationship between ART use and virologic suppression [41, 42], there is need for intensification of ART adherence support among YLWH facing adherence challenges to avoid viral rebound, virologic failures or the development of HIV drug resistance, especially if routine viral load monitoring is not readily available. The adherence approach should follow the caring, respectful and compassionate model of care [43]. This approach is particularly important for YLWH since they are more likely to be lost from care [12] for instance if it appears they are being censured for missed ART doses during the adherence support sessions. Additionally, barriers to optimal ART adherence among YLWH should be identified and addressed at an individual level. Some of the commonly reported barriers among YLWH include sub-optimal disclosure of HIV status [44, 45], forgetfulness or being busy with different activities [46], anticipated HIV-related stigma [44, 46], psychological distress [46, 47], substance use [46, 48], inadequate social support and lower medication self-efficacy [49]. Beyond VNS, sub-optimal ART adherence may eventually result in ART resistance problems and this can compromise available and future ART options [50].

To the best of our knowledge, this is the first study from SSA to report a significant association between low BMI (being underweight relative to normal body weight) and higher odds of VNS among YLWH. Similar associations have been reported in studies involving the general adult population with HIV [51–53]. Contrast to our finding, a study conducted in Northeast Ethiopia [40] found a null association. At the Kenyan coast, where the levels of inequality and poverty are high [54], the undernutrition observed among YLWH (30% were underweight) could be a proxy marker for inadequate access to foods rich in nutrients due to lack of resources. ART medications are strong and individuals on ART are often required to take highly nutritious food (e.g., up to five servings a day is recommended in our setting) but in the absence of means to afford such nutritious food, instances of YLWH taking ART on an empty stomach, missing some doses or not taking it at all (hence poor adherence) have been reported [55, 56]. As such, poor viral suppression is expected. However, in the inverse direction, it is plausible that persistent VNS can lead to body wasting hence lower BMI. Therefore, the direction of the association between BMI and VNS can only be discerned by studies of longitudinal design. Nonetheless, to overcome any poverty-related ART adherence obstacle in our setting, nutritional support programmes facilitated by the government or other donors and run through the HIV clinics are recommended. Nutritional counselling should be promoted at the HIV clinics, educating YLWH about food types with the most benefit to their body while on ART but at the same time considering the cost implications.

In this study, rural residency was significantly associated with nearly twofold higher odds of VNS. This finding is difficult to compare within the literature of virologic outcomes in YLWH; we are not aware of any study that reports this kind of association. However, the finding is supported by an investigation from Ethiopia involving adults living with HIV [57]. Living in a rural area could be an indirect marker of treatment interruption because of reasons such as difficulties in accessing HIV treatment services. In remote areas of Kenya, healthcare services are often inaccessible as health facilities are a distant away and roads leading into such facilities are often in bad condition, attracting high transport costs [58]. The high transport costs may be a deterrent to YLWH in seeking routine HIV care, especially if the financial obligation lies with them. Treatment interruption is significantly associated with poor virological and immunological outcomes [57].

We did not observe any significant associations between VNS and demographic variables of age and sex. In contrast, other studies have found significant association between VNS and youth age [22–24] or sex [24, 59]. In support of our finding, an Ethiopian study [23] found a null association between VNS and participant sex. We think that the lack of association in our study may be because these young people aged 18 to 24 years are at a developmental age where they are equally exposed to the challenges of transitioning into adulthood while living with HIV, regardless of gender. For instance, as they are now considered adults by law and society, it is expected that they take charge of aspects of individual health including ART taking (without close monitoring anymore) and honoring clinic appointments for ART refill or regular check-up.

HIV-related stigma has been associated with higher odds of VNS in YLWH [25] but this association was not replicated in our study. Furthermore, no associations were observed between VNS and any of the HIV clinical factors including ART regimen, duration on ART and WHO clinical staging (at either the univariate or multivariable analyses). Presence of opportunistic infection was significantly associated with VNS at the univariate but not multivariable analyses. These findings generally contrast findings from studies involving YLWH [22–24] but compares to those from previous reports involving general adults living with HIV [40, 52]. The range and level of care that these YLWH receive at the HIV clinics may be a mitigating factor, over 90% of them self-reported being satisfied with the care they received at their point of care.

The findings reported in this study should be interpreted in the context of several study limitations. First, the cross-sectional analyses do not allow for any causal inference. Second, we did not include underlying HIV drug resistance in the analyses, and this could also be a cause of VNS [60]. Future analysis of data from this sub-population will help arbitrate the contribution of HIV drug resistance to VNS in coastal Kenya, aside from suboptimal ART adherence. This line of enquiry will be particularly important in the minimization of unnecessary/premature ART regimen switch—often to limited and expensive options [60]. Third, data on ART adherence was based on self-report, which may be affected by recall and social desirability bias. Fourth, study findings may not be generalizable to YLWH outside the study context. Relatedly, even within the study context, caution should be exercised in generalizing findings to all YLWH since we only recruited participants from public HIV facilities and used consecutive sampling method. Some YLWH could be accessing services exclusively from private facilities offering HIV-related services.

## Conclusion

A considerable proportion of YLWH in this study were virally non-suppressed. In this setting, rural residency, being underweight and poor ART adherence are important indicators associated with VNS that necessitate intensified adherence counselling and nutritional support and education which should target YLWH mostly from rural areas. Given the high frequency of VNS, there is need to closely monitor viral load and profile HIV drug resistance patterns in youths from the Kenyan coast with confirmed virologic failure. The latter will help understand whether drug resistance also contributes to poor viral suppression in addition to, or exclusive of suboptimal ART adherence but also inform the need for ART regimen switching.

## Data Availability

The dataset and associated files used for analysis of this study are available in Harvard dataverse at https://doi.org/10.7910/DVN/GUMGBW. Application for access can be made through the data governance committee of the KEMRI Wellcome Trust Research Programme (KWTRP) who will review the application and advise as appropriate ensuring that uses are compatible with the consent obtained from participants for data collection. Requests can be sent to the coordinator of the Data Governance Committee using the following email: dgc@kemri-wellcome.org.

## Abbreviations

ACASI: Audio computer assisted self-interview
ART: Antiretroviral therapy
BMI: Body mass index
MGLS: Morisky, Green and Levine Medication Adherence Scale
PHQ-9: 9 Item patient health questionnaire
SSA: Sub-Saharan Africa
VNS: Virological non-suppression
WHO: World Health Organization
YLWH: Young people living with HIV
